# Effects of lamivudine on serum albumin levels correlate with pretreatment HBV-DNA levels in cirrhotic patients

**DOI:** 10.1186/1476-5926-6-3

**Published:** 2007-05-01

**Authors:** Makoto Nakamuta, Kazuhiro Kotoh, Munechika Enjoji, Eiji Kajiwara, Junya Shimono, Akihide Masumoto, Toshihiro Maruyama, Norihiro Furusyo, Hideyuki Nomura, Hironori Sakai, Kazuhiro Takahashi, Koichi Azuma, Shinji Shimoda, Yuichi Tanabe, Jun Hayashi

**Affiliations:** 1 Department of Medicine and Bioregulatory Science, Graduate School of Medical Sciences, Kyushu University, Japan; 2 Department of Medicine, Fukuoka City Hospital, Fukuoka, Japan; 3 Department of Internal Medicine, Nippon Steel Yawata Memorial Hospital, Kitakyushu, Japan; 4 Department of Medicine, Yahata Saiseikai Hospital, Kitakyushu, Japan; 5 Department of Clinical Research, National Hospital Organization Kokura Hospital, Kitakyushu, Japan; 6 Department of Medicine, Kitakyushu Municipal Medical Center, Kitakyushu, Japan; 7 Department of Environmental Medicine and Infectious Diseases, Graduate School of Medical Sciences, Kyushu University, Japan; 8 Department of Internal Medicine, Shin-Kokura Hospital, Kitakyushu, Japan; 9 Department of Gastroenterology, National Hospital Organization Kyushu Medical Center, Fukuoka, Japan; 10 Department of Medicine, Hamanomachi Hospital, Fukuoka, Japan; 11 Department of Medicine and Clinical Science, Graduate School of Medical Sciences, Kyushu University, Japan; 12 Department of Medicine and Biosystemic Science, Graduate School of Medical Sciences, Kyushu University, Japan

## Abstract

**Background:**

Lamivudine treatment has been recently demonstrated to increase the serum albumin levels in cirrhotic patients with hepatitis B virus (HBV) infection, but the precise mechanism remains unclear. We hypothesized that the improvement of hypoalbuminemia by lamivudine may be attributable to the reduction of HBV replication itself, rather than to cessation of hepatitis. In order to confirm this hypothesis, in this study we evaluated factors which correlated with the increase in serum albumin levels. Fifty-four patients (Child-Pugh A/B/C, 35/9/10) with HBV-related liver cirrhosis who had been treated with lamivudine for more than 12 months were evaluated. We analyzed the correlation between the increase in serum albumin levels at month 12 after starting treatment (Δ-albumin) and various pretreatment variables. We also analyzed the correlation between Δ-albumin and the reduction in serum levels of HBV-DNA (Δ-HBV-DNA) or alanine aminotransferase (Δ-ALT) at month 12.

**Results:**

The average Δ-albumin was 0.38 g/dL and only serum HBV-DNA levels before treatment correlated significantly with Δ-albumin. We also analyzed the correlation in patients whose alanine aminotransferase levels were normalized after 12 months so that the possible influence of breakthrough hepatitis could be excluded. Even among this subgroup of patients, there was no significant correlation between Δ-albumin and either pretreatment alanine aminotransferase levels or Δ-ALT. In contrast, in patients whose serum HBV-DNA was undetectable at month 12, we found a significant correlation between Δ-albumin and both pretreatment serum HBV-DNA levels and Δ-HBV-DNA.

**Conclusion:**

Our results demonstrated that albumin levels are associated with pretreatment HBV-DNA but not with alanine aminotransferase levels.

## Background

Chronic hepatitis B is an important cause of morbidity and mortality resulting from cirrhosis-related liver failure and hepatocelluar carcinoma (HCC) [1-3]. Lamivudine, a nucleoside analogue with potent antiviral effects against hepatitis B virus (HBV), has been shown to be effective both in patients with chronic hepatitis and also those with liver cirrhosis [4-6]. In cirrhotic patients, decreased HBV-DNA loads following lamivudine treatment result in decreased serum levels of alanine aminotransferase (ALT), increased serum albumin levels, and improvement of the Child-Pugh score [7-13]. The underlying mechanism for the increase in albumin levels after lamivudine treatment has not been determined. It has been suggested that the improvement of hypoalbuminemia may be attributable to the cessation of hepatic inflammation. However, earlier treatments such as glycyrrhizin, ursodeoxycholic acid [14,15], predonisolone [16], and Stronger Neo-Minophagen C therapy [17], all of which reduce ALT levels in viral cirrhotic patients, do not result in improvement of hypoalbuminemia. Furthermore, it has been shown that there is no significant correlation between serum ALT levels and HBV-DNA loads in patients with HBV [18-20]. We hypothesized that the improvement of hypoalbuminemia by lamivudine may be attributable to the reduction of HBV replication itself, rather than to cessation of hepatitis. In order to confirm this hypothesis, we evaluated several laboratory parameters in cirrhotic patients treated with lamivudine that could influence serum albumin levels.

## Results

**Figure 1:**
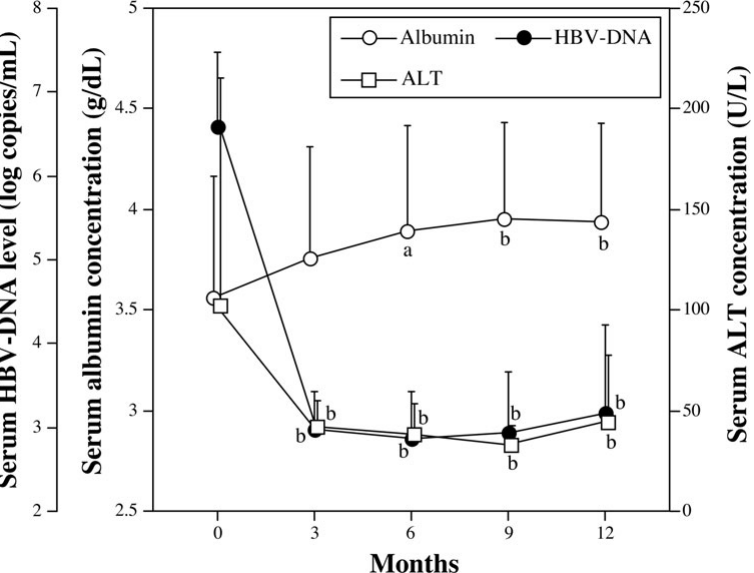
**Time course of albumin, HBV-DNA, and ALT levels in lamivudine treatment**. The average serum levels of albumin (closed circles), HBV-DNA (open squares), and ALT (open circles) at 3-month intervals from the start of lamivudine therapy are plotted. Soon after the start of treatment, serum albumin levels increased rapidly and simultaneously with a decrease in HBV-DNA and serum ALT levels. The data represent mean + SD (a, b; *p *< 0.05 and *p *< 0.01 vs. 0 month, respectively).

Fifty-four cirrhotic patients with HBV infection were analyzed (Table 1, see Materials and methods). Before the treatment, there was no significant correlation between either serum ALT or albumin levels and HBV-DNA loads in our patients (data not shown). Following lamivudine treatment, the levels of HBV-DNA and ALT rapidly decreased while albumin levels simultaneously increased (Figure 1). HBV-DNA levels decreased significantly from 6.59 ± 0.18 log copies/mL to 2.98 ± 0.12 log copies/mL at 3 months after treatment (*p *< 0.01), and decreased further to 2.87 ± 0.14 log copies/mL and 2.94 ± 0.18 log copies/mL at 6 and 9 months, respectively. Similarly, ALT levels also decreased significantly from 102.1 ± 10.4 U/L to 42.0 ± 2.7 U/L at 3 months after treatment (*p *< 0.01), and to 38.8 ± 4.1 U/L and 33.1 ± 2.4 U/L at 6 and 9 months, respectively. However, at 12 months there was a slight increase in both HBV-DNA and ALT levels (3.17 ± 0.21 log copies/mL and 44.3 ± 8.6 U/L, respectively), although the differences between values at 9 and 12 months were not statistically significant. The serum levels of albumin increased from 3.56 ± 0.09 g/dL to 3.76 ± 0.08 g/dL at 3 months after treatment, and increased further to 3.89 ± 0.08 g/dL (*p *< 0.05) and 3.95 ± 0.08 (*p *< 0.01) g/dL at 6 and 9 months, respectively. At 12 months, albumin levels remained steady at 3.94 ± 0.08 g/dL.

**Table 1:** Characteristics of the patients

	Child A	Child B	Child C	Total
*n*	35	9	10	54
Male/female	26/9	7/2	5/5	38/16
Age	53.0 ± 9.1	54.9 ± 4.6	49.5 ± 9.1	52.6 ± 8.8
Albumin (g/dL)	3.85 ± 0.43	3.12 ± 0.38	2.94 ± 0.57	3.56 ± 0.6
Bilirubin (mg/dL)	0.90 ± 0.43	1.25 ± 0.35	3.09 ± 1.28	1.37± 1.07
ALT (U/L)	118.2 ± 125.5	62.7 ± 43.2	80.6 ± 96.8	102.0 ± 113.0
Platelet (× 10^4^/μL)	11.8 ± 5.3	7.3 ± 3.2	6.3 ± 2.5	10.0 ± 5.2
HBeAg (+/-)	17/18	6/3	6/4	29/25
HBV-DNA (log copies/mL)				
< 5.0	1	1	2	4
5.0 ≤ x < 7.0	21	4	3	28
≥ 7.0	13	4	5	22

To identify the factors associated with increased serum albumin levels, correlations between the increase in serum albumin levels at 12 months after the start of treatment (Δ-albumin) and basic variables before treatment were examined using the data for all patients. In this analysis, only HBV-DNA load correlated significantly with Δ-albumin (t = 2.66, r^2 ^= 0.120089, *p *= 0.0103), whereas age, sex, HBeAg, ALT, bilirubin, platelet count, and Child-Pugh classification did not (Table 1).

**Figure 2:**
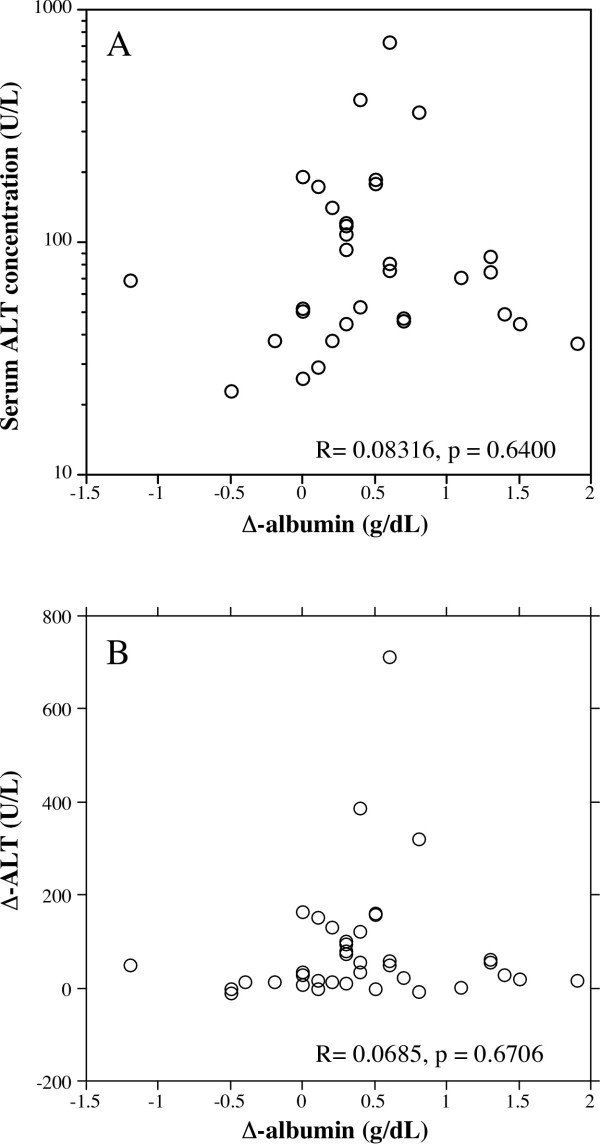
**Correlation between ALT levels before treatment and Δ-albumin (A), and Δ-ALT and Δ-albumin (B)**. In patients whose serum ALT levels were normalized at 12 months after treatment, there was no significant correlation Δ-albumin and pretreatment serum ALT levels (A). There was also no significant correlation Δ-albumin and Δ-ALT (B).

Although we found no correlation between Δ-albumin and pretreatment serum ALT levels for the entire patient population, the possibility remained that breakthrough hepatitis or continuous elevation of ALT might interfere with Δ-albumin. Indeed, two patients showed breakthrough hepatitis, where ALT levels increased to over 100 U/L, and 20 patients still showed abnormally high ALT (> 35 U/L) at 12 months after treatment. We next evaluated the correlation between Δ-albumin and pretreatment serum ALT levels among the 32 patients in whom serum ALT levels were normalized (< 35 U/L) at 12 months after the start of therapy. As shown in Figure 2A, there was no significant correlation between Δ-albumin and pretreatment serum ALT levels in this subgroup of patients (*r *= 0.083, *p *= 0.64). We also evaluated the correlation between Δ-albumin and reduction in ALT levels at month 12 after starting treatment (Δ-ALT) in this group, but there was still no significant correlation between Δ-albumin and Δ-ALT (*r *= 0.0685, *p *= 0.67) (Figure 2B).

**Figure 3:**
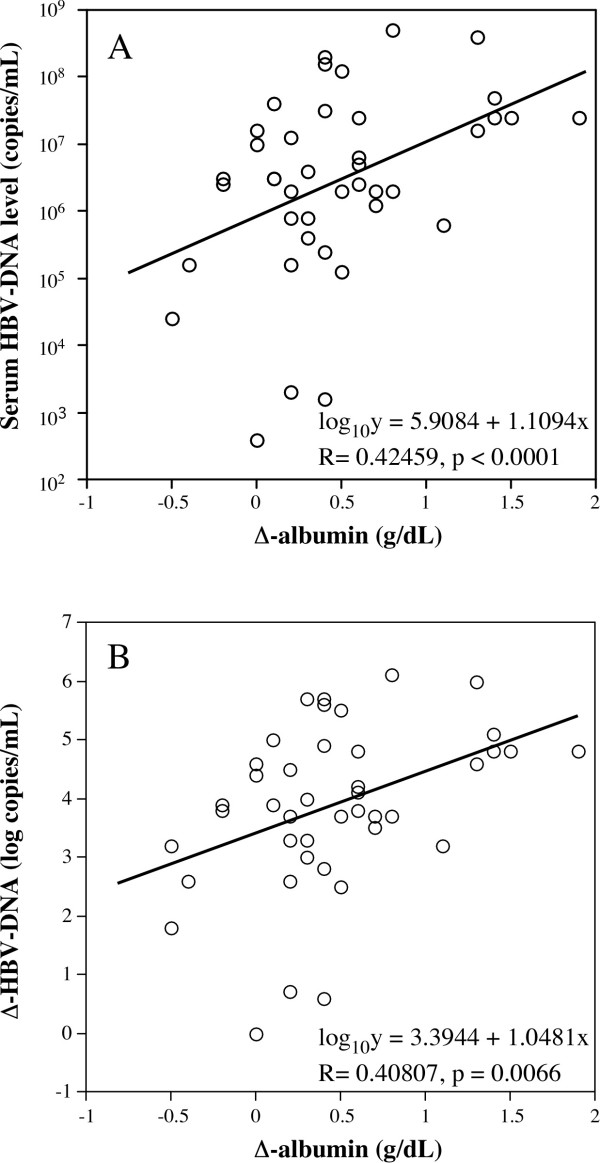
**Correlation between HBV-DNA levels before treatment and Δ-albumin (A), and Δ-HBV-DNA and Δ-albumin (B)**. Patients whose serum HBV-DNA was undetectable at 12 months after treatment, there was a significant correlation between Δ-albumin and both pretreatment serum HBV-DNA levels (A) and Δ-HBV-DNA (B).

Furthermore, we evaluated the correlation between Δ-albumin and serum HBV-DNA levels before treatment among the 41 patients in whom serum HBV-DNA levels were undetectable at 12 months post-treatment. In this analysis, we found a significant correlation between Δ-albumin and the serum levels of HBV-DNA before the start of therapy (*r *= 0.42459, *p *< 0.0001) (Figure 3A). We also evaluated the correlation between Δ-albumin and reduction in HBV-DNA levels at month 12 after starting treatment (Δ-HBV-DNA) in this group, and we again found that Δ-albumin significantly correlated with Δ-HBV-DNA (*r *= 0.40807, *p *= 0.0066) (Figure 3B).

## Discussion

This study demonstrated the followings: 1) HBV-DNA, but not ALT levels, before lamivudine treatment was associated with increased serum albumin levels at 12 months after treatment (Δ-albumin); 2) Even among those patients who showed cessation of hepatitis following treatment, there was no correlation between either pretreatment ALT levels or Δ-ALT and Δ-albumin; 3) In contrast, in the analysis of subjects with undetectable HBV-DNA levels after treatment, there was significant correlation between both pretreatment HBV-DNA levels and Δ-HBV-DNA and Δ-albumin. Taken together, these results suggest that the improvement of hypoalbuminemia following lamivudine treatment is attributable to a reduction of HBV replication, but not to cessation of hepatitis.

We do not deny the idea that cessation of hepatitis, which is represented by lowering of serum ALT levels, contributed to and increase of serum albumin levels. In true, we think that replicative HBV and inflammation are closely related; however, in our study, HBV reduction statistically showed more effect improving serum albumin levels than decreasing the inflammation marker ALT. This may happen perhaps because, in cirrhotic patients, fibrosis is the main pathological change (compared with inflammation), and the correlation between, on the one hand, serum albumin or HBV-DNA levels and, on the other hand, ALT levels was in some degree weakened as the cirrhotic change proceed. Therefore, in cirrhotic patients, Δ-ALT is within a narrower range and ALT levels cannot influence albumin levels significantly.

How does lowering of HBV load induces the increase of albumin levels in an inflammation-independent manner? Hui et al. [7] recently showed that emergence of phenotypic resistance of HBV-DNA was associated with a rapid decline in serum albumin levels following prolonged lamivudine treatment, although they did not report whether a correlation existed between serum ALT levels and serum HBV loads. In a series of studies in woodchucks and Hep G2 cells, Kosovsky et al. demonstrated that HBV replication inversely correlated with cell proliferation and DNA synthesis by hepatocytes [21-23]. Yang et al. has analyzed gene expression profiles of HepG2 cells with or without HBV [24]. However, whether HBV replication directly influences the ability of infected hepatocytes to synthesize protein is still unclear and further studies are needed.

Our results indicate that increased serum albumin levels should be expected in cirrhotic patients following lamivudine treatment, and that this occurs independently of serum ALT levels and Child-Pugh's score before treatment, as shown by the lack of a correlation between those variables and Δ-albumin. Previous studies of lamivudine treatment for liver cirrhosis showed that fatalities occur because of acute liver failure after discontinuation of lamivudine [25,26] or emergence of lamivudine-resistance mutants [27,28]. Recent reports, however, indicate that prolonged use of lamivudine for cirrhotic patients is safe and effective [5,29,30]. Furthermore, since adefovir is effective for treating resistant mutants [31-33], lamivudine therapy should be encouraged. Hypoalbuminemia, which causes ascites, edema, and hydrothorax, lowers the quality of life of cirrhotic patients [34,35]. High viral load of HBV is associated with higher mortality and morbidity in cirrhotic patients in consequence of high occurrence or recurrence rate of HCC [36,37]. Lamivudine is effective for preventing or delaying occurrence of liver failure and HCC through lowering HBV, and therefore can be a first choice drug for patients with high HBV levels regardless of serum ALT levels.

## Methods

### Patients

A total of 54 cirrhotic patients with HBV infection were evaluated, including 38 males and 16 females, ranging in age from 28 to 71 years (mean 52.6 years) (Table 1). Informed consent was obtained from each patient prior to their entering the study. Liver cirrhosis was diagnosed based on liver biopsy (n = 11), laboratory data, ultrasonography, and/or computed tomography. Patients were classified as Child-Pugh class A, B and C (35, 9, and 10 patients, respectively). For all patients, the existence of serum HBV-DNA was confirmed by TMA assay (10^3.7^–10^8.7 ^genome equivalents/mL; 3.7–8.7 log genome equivalents [LGE]/mL) (Chugai Diagnostic Science, Tokyo, Japan) or by a Roche Monitor kit (10^2.6^–10^7.6 ^copies/ml; 2.6–7.6 log copies/mL) (Roche Diagnostics, Tokyo, Japan) before treatment. HBe-Ag was positive in 29 patients and negative in 25 patients. Patients with fatty liver, viral hepatitis C, a history of alcohol abuse, or autoimmune disorders such as autoimmune hepatitis and primary biliary cirrhosis were excluded. None of the patients had a prior history of treatment for hepatocellular carcinoma.

Patients had been treated with lamivudine (100 mg, once a day) without interruption for more than twelve months at Kyushu University Hospital and its affiliated hospitals. Basic laboratory data, such as platelet counts, serum ALT levels, bilirubin, albumin, serum HBV-DNA load (Roche Monitor kit: Roche Diagnostics) and HBe-Ag were determined at least every 3 months.

### Statistical analysis

Data are expressed as mean ± SD, and statistical comparisons were performed using chi-squared test for categorical data and one-way ANOVA for numeric data. In cases where the serum HBV-DNA load was less than 2.6 log copies/mL, it was entered as 2.6 log copies/mL. For the analysis of correlations between two continuous variables, a simple regression model was used. For the analysis of discontinuous variables, such as sex and HBe-Ag, statistical differences were confirmed using Mann-Whitney U test or Kruskal-Wallis test.

## Competing interests

The author(s) declare that they have no competing interests.

## Authors' contributions

MN and ME participated in the experimental design and writing of the manuscript. JH participated in the experimental design. KK performed most of the analysis. YT, EK, JS, AM, TM, NF, HN, HS, KT, KA, and SS collected and supplied the clinical data of patients.

**Table 2:** Correlations between Δ-albumin and basic variables before treatment

	t	R^2^	P-value
Age	-0.14	0.000398	0.8873
ALT	0.67	0.008536	0.5064
Bilirubin	-0.04	0.000036	0.9659
Platelet	-0.87	0.014279	0.3894
HBV-DNA	2.66	0.120089	0.0103
HBeAg (+/-)	-	-	0.6201
Sex (male/female)	-	-	0.4251
Child-Pugh's classification	-	-	0.0968
